# Assessment of the equivalent dipole layer source model in the reconstruction of cardiac activation times on the basis of BSPMs produced by an anisotropic model of the heart

**DOI:** 10.1007/s11517-017-1715-x

**Published:** 2017-11-13

**Authors:** Arno M. Janssen, Danila Potyagaylo, Olaf Dössel, Thom F. Oostendorp

**Affiliations:** 1 The Netherlands Heart Institute, Utrecht, The Netherlands; 2 The Donders Institute for Brain, Cognition and Behaviour, Radboud University Medical Centre, Nijmegen, The Netherlands; 3 The Institute of Biomedical Engineering, Karlsruhe Institute of Technology (KIT), Karlsruhe, Germany

**Keywords:** Equivalent dipole layer, Forward and inverse problems of ECG, Activation times imaging, Influence of anisotropy, Fastest route algorithm

## Abstract

Promising results have been reported in noninvasive estimation of cardiac activation times (AT) using the equivalent dipole layer (EDL) source model in combination with the boundary element method (BEM). However, the assumption of equal anisotropy ratios in the heart that underlies the EDL model does not reflect reality. In the present study, we quantify the errors of the nonlinear AT imaging based on the EDL approximation. Nine different excitation patterns (sinus rhythm and eight ectopic beats) were simulated with the monodomain model. Based on the bidomain theory, the body surface potential maps (BSPMs) were calculated for a realistic finite element volume conductor with an anisotropic heart model. For the forward calculations, three cases of bidomain conductivity tensors in the heart were considered: isotropic, equal, and unequal anisotropy ratios in the intra- and extracellular spaces. In all inverse reconstructions, the EDL model with BEM was employed: AT were estimated by solving the nonlinear optimization problem with the initial guess provided by the fastest route algorithm. Expectedly, the case of unequal anisotropy ratios resulted in larger localization errors for almost all considered activation patterns. For the sinus rhythm, all sites of early activation were correctly estimated with an optimal regularization parameter being used. For the ectopic beats, all but one foci were correctly classified to have either endo- or epicardial origin with an average localization error of 20.4 mm for unequal anisotropy ratio. The obtained results confirm validation studies and suggest that cardiac anisotropy might be neglected in clinical applications of the considered EDL-based inverse procedure.

**Electronic supplementary material:**

The online version of this article (10.1007/s11517-017-1715-x) contains supplementary material, which is available to authorized users.

## Introduction

The electrocardiogram (ECG) is an important diagnostic tool in cardiology. The ECG is the result of electrical currents generated by the myocardial cells that initiate and accompany the contraction of the human heart. Based on the ECG, a cardiologist can assess the patient’s heart condition, but even for experienced clinicians, it is difficult to accurately translate the information obtained from the ECG to cardiac electrograms. The electromagnetic fields originating from electrical activity of the heart are attenuated in the body volume and only low-resolution information content can be detected on the thorax surface. The goal of non-invasive electrocardiographic imaging (ECG imaging) [35, 41], also called the inverse problem of electrocardiography, is to reconstruct high-resolution maps of cardiac electrical activity from the signals recorded at the body surface (either 12-lead ECGs or multi-lead Body Surface Potential Maps). Despite the progress made in the methodology of ECG imaging over the past decades and the promising application of the technique in clinical studies [3, 39, 48], an accurate estimation of cardiac sources remains a challenging problem.

Two popular surface source models are the epicardial potential model [2, 38, 42] and the equivalent dipole layer (EDL) model [9, 12, 13, 36]. In the former model, the current sources inside the heart are replaced by potential sources on a surface that encompasses the whole heart (the pericardium). The EDL model represents the sources by a current dipole layer on the whole myocardial surface, including inner cavities. This enables the possibility to distinguish between endocardial and epicardial activation. In the work presented here, the EDL source model is used in the inverse procedure.

According to the EDL theory, under certain assumptions, the potential generated by the three-dimensional electric source distribution within the myocardium is equal to the potential generated by a dipole layer on the myocardial surface. The strength of the layer is proportional to the amplitude of transmembrane potential (TMP) upstroke on this surface. In our implementation, the TMP is parameterized by the depolarization and repolarization times, which allows the direct computation of these parameters from the BSPMs (see Section 2 for details). The depolarization times, also called activation times (AT), provide spatiotemporal features of the propagating wave front within the heart that can be used for guiding ablation procedures [8].

However, the equivalence between the electrical sources within the myocardium and the appropriate dipole layer on the heart surface is only valid under the appropriate assumptions about the electrical conductivities inside the myocardial volume. The equivalence of the EDL model and the three-dimensional sources was first shown to be valid in a bidomain model with two interpenetrating continuous media in the myocardial volume: intra- and extracellular domains with isotropic conductivities [15]. Thereafter, the applicability of the EDL source model was extended to anisotropic conductivities as long as the anisotropy ratio, i.e., the ratio between the conductivity along and perpendicular to the myocardial fibers, is the same for the intra- and the extracellular media [14]. Although there is no clear-cut consensus about the values of the intra- and extracellular conductivities [24, 40, 43], the assumption of equal anisotropy ratios in these spaces does not reflect the reality. From previous studies, it is known that the excitation wavefront, which is characterized by the TMPs, and therewith associated potential distributions in the body volume conductor are strongly affected by the fiber orientation and heart anisotropy [4, 45]. On the other hand, the EDL-based model has claimed successful results in several studies on ECG imaging [21, 30, 31], demonstrating potenial power of this noninvasive technique in reconstructing endocardial and epicardial activation patterns and identifying the early sites of ventricular excitation [33]. Furthermore, the EDL model also proved its merit in simulation of intracardiac electrograms [1, 19], which implies it to be a valid approximation for clinical applications.

The purpose of the present study is to quantify the errors that result from using the EDL approximation, as developed in Nijmegen, The Netherlands [6], for noninvasive imaging of BSPMs generated for a range of anisotropy ratios in the heart conductivity tensors. For this purpose, the inverse was applied to realistic simulation data for different excitation patterns (sinus rhythm and ectopic foci) produced by a highly detailed propagation and volume conductor models, as developed in Karlsruhe, Germany [44], that take myocardial anisotropy properties into account. To keep the study more realistic, the inverse analysis was performed blindly.

**Fig. 1:**
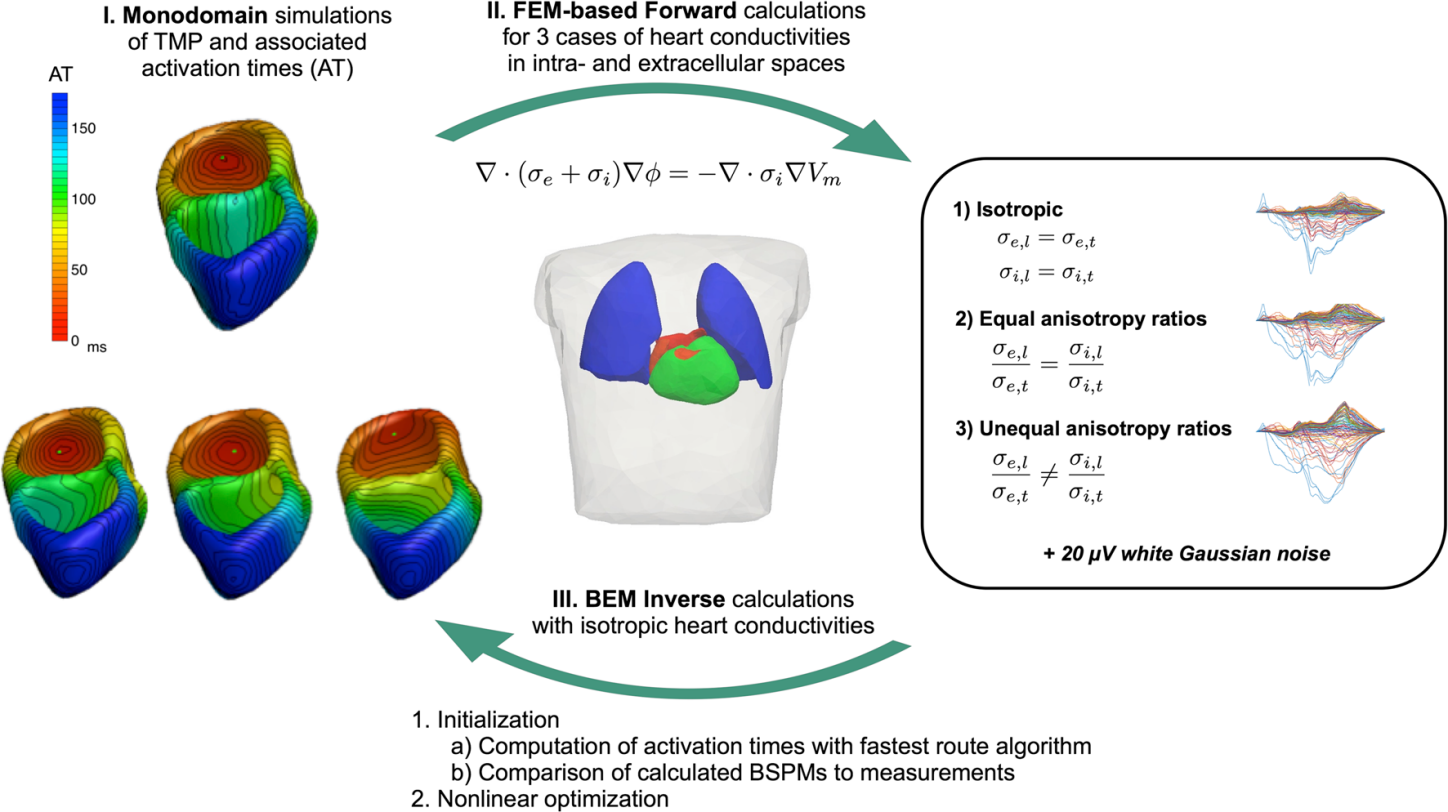
Workflow diagram used for this study: At the first step, the TMP are simulated on a voxel grid with a monodomain solver. Afterwards, the obtained transmembrane potentials are interpolated onto a tetrahedral mesh, and forward calculations for three cases of cardiac conductivity setups are performed. Finally, the EDL-based inverse procedure is applied to the three cases of simulated BSPMs

The simulation workflow employed in the present study is visualized in Fig. 1.

## Methods

An anisotropic monodomain model developed at the Karlsruhe Institute of Technology, Germany [44], was used to compute the transmembrane potentials (TMPs) throughout the myocardium during ventricular activation. The TMPs corresponding to activation sequences of sinus rhythm and eight ventricular ectopic foci (septal, right and left ventricular, including endocardial as well as epicardial ectopic origins) were generated. The obtained TMPs were subsequently used to compute electrocardiograms at 120 electrode positions in a finite element volume conductor model. These body surface potential maps were calculated for three simulation cases, in which the intra- and extracellular conductivity anisotropy ratios of the myocardium were set to be:

1. One — isotropic case
2. Equal — equal anisotropy ratio case
3. Unequal — unequal anisotropy ratio case

In the isotropic case, the only source of errors in the EDL-inverse (in this model study) is the intrinsic ill-posedness of the inverse problem. This allows the separation of this type of error from those caused by neglecting cardiac anisotropy.

The constructed BSPMs were used as input for the EDL-based inverse procedure developed at the Radboud University Medical Center in Nijmegen, the Netherlands [6]. This EDL-based inverse method is a nonlinear optimization routine reconstructing the AT distribution, given a template for temporal course of the TMP. The same geometrical model as in the Karlsruhe computations was used, but always with isotropic bulk conductivity for the myocardium. The activation patterns on the myocardial surface found by the EDL-based non-linear inverse routine were compared to the AT distributions extracted from the results of the anisotropic monodomain model calculations. The true AT patterns were not disclosed to the researchers that performed the inverse computations.

### Volume conductor models

An inhomogeneous volume conductor model (VCM) was created, based on thoracic magnetic resonance images (MRI) of a 27-year old healthy volunteer. The torso MRI data had a voxel size of 1 × 1 × 2 mm^3^, while the heart was imaged with the resolution of 1 × 1 × 1 mm^3^. The segmentation was performed in a semi-automatic manner using region growing and active contours methods. The model included lungs, blood filled cavities within the heart, thorax, and ventricles. The same surface geometries were used for both forward calculations with the finite element method (FEM) and inverse calculations with the boundary element method (BEM). For the forward FEM calculations, the tetrahedral VCM contained 791983 nodes, including 48671 ventricular nodes. The BEM-based inverse computations were performed with a VCM of which the surface meshes for all tissue types were extracted from the FEM model and then simplified by removing the vertices and rebuilding the faces. This mesh simplification resulted in a VCM with 5877 nodes, including 1500 ventricular and 1002 torso nodes. As the node locations were preserved, the BEM-model nodes were a subset of the points within the FEM grid.

For all tissues in the VCM, the conductivity values can be found in Table 1. The bulk conductivity of the myocardium in the BEM model was set up to be the sum of intra- and extracellular conductivities from the isotropic FEM model. Isotropic conductivity (i.e. anisotropic ratio of 1) means that the conduction is equal along (longitudinal) and perpendicular (transversal) to the myocardial muscle fibers. For the equal anisotropy ratio cases, the longitudinal conductivities were set to be three times of the transversal ones, in both extra- and intracellular domains. For unequal anisotropy, the ratio between conductivity components in longitudinal and transversal directions was assigned to be 3 and 9 in extra- and intracellular domains, respectively.

**Table 1:** Conductivity values used

Tissue type	Conductivity [S m^− 1^]
Thorax	0.2
Lungs	0.04
Blood	0.6
Heart BEM	0.2
Heart FEM isotropic	*σ*_*e*,*t*_ = *σ*_*e*,*l*_ = 0.15
	*σ*_*i*,*t*_ = *σ*_*i*,*l*_ = 0.05
Heart FEM equal anisotropy ratio	*σ*_*e*,*l*_ = 0.45
	*σ*_*i*,*l*_ = 0.15
	*σ*_*e*,*l*_/*σ*_*e*,*t*_ = *σ*_*i*,*l*_/*σ*_*i*,*t*_ = 3
Heart FEM unequal anisotropy ratio	*σ*_*e*,*l*_ = 0.45
	*σ*_*i*,*l*_ = 0.45
	*σ*_*e*,*l*_/*σ*_*e*,*t*_ = 3
	*σ*_*i*,*l*_/*σ*_*i*,*t*_ = 9

The conductivity values for thorax, lungs and blood are based on Gabriel et al. [11]. The heart conductivity values for the unequal anisotropy ratio are in agreement with Colli Franzone et al. [5]. The values for the isotropic and equal anisotropy cases are based on the transversal direction values from the unequal anisotropy ratio case. The bulk conductivity of the heart in the BEM model is set up to be the sum of intra- and extracellular conductivities from the isotropic FEM model. *σ*_*e*,*l*_ and *σ*_*i*,*l*_are the conductivities in longitudinal and transversal directions for extra- and intracellular spaces, respectively; *σ*_*e*,*t*_ and *σ*_*i*,*t*_ are the conductivities in longitudinal and transversal directions for extra- and intracellular spaces respectively

### Forward calculations

In order to simulate TMP distributions for sinus rhythm and eight ventricular ectopic foci, the ventricular cell model proposed by Ten Tusscher and Panfilov [46] was used. The cardiac excitation propagation was computed with the parallel monodomain solver acCELLerate [44] on a ventricular voxel grid (0.4 mm resolution). A rule-based approach was used for creation of the fiber orientation [24]. Different heterogeneities for calculation of the TMP distributions were integrated into the model according to [49].

For a normal sinus rhythm, a rule-based endocardial stimulation profile imitating the Purkinje fibers was used [23, 24]. For simulating ectopic beats, a spherical area with a two voxels radius was stimulated in order to initiate excitation. The locations of the foci origins were chosen in a pairwise manner: an epicardial focus was accompanied by an endocardial one, projected through the myocardial wall. The extrasystoles considered in this study listed: two foci on both sides of the septal wall, two left ventricular free wall foci, two foci on the right ventricular free wall, and two beats originating from a basal part of the ventricles close to the septal wall.

The TMP distributions were interpolated from the voxel grid to the ventricular tetrahedral mesh. Using the bidomain equations and FEM [24], the electric potentials within the whole VCM were computed for each millisecond during the depolarization phase. For each activation sequence (sinus rhythm and eight ventricular ectopic foci), the potentials were extracted at 120 electrode positions uniformly distributed over the body surface. As the node locations were preserved during the conversion of the FEM-model to the BEM-model, the electrode positions in the inverse calculations were the same. For the inverse calculations, the forward simulated BSPMs were contaminated with 20 *μ* *V* Gaussian white noise.

### Inverse activation estimation

The current source that generates the ECG is a three-dimensional distributed dipole density **J** ^*i*^(**r**) throughout the myocardium. The strength of this current source is proportional to the gradient of the transmembrane potential *ϕ*_*h*_(**r**):

1 $$ \mathbf{J}^{i}(\mathbf{r}) = -\sigma_{i} (\mathbf{r}) \nabla \phi_{h}(\mathbf{r}) $$

where *σ*_*i*_ is the intracellular conductivity tensor [15]. Geselowitz showed [14] that under the condition that the anisotropy ratio is equal for the intra- and extracellular myocardium, this current source can be replaced by an equivalent dipole layer on the myocardial surface (encompassing both epicardium and endocardium) that is directed perpendicular to the surface and whose strength is proportional to the transmembrane potential on the surface. This is the source that is used in the EDL-model.

During the depolarization phase, the transmembrane potential can be considered to be either “at rest” or “activated” [22]. Consequently, the equivalent source is completely described by the activation times *τ*(**r**) on the myocardial surface. Then, the potential at observation point **r** ^∗^on the body surface at time *t* follows from

2 $$ \phi (\mathbf{r}^{*},t) = {\int}_{S_{h}} H(t-\tau({\mathbf{r}})) A(\mathbf{r},\mathbf{r}^{*}) \: dS(\mathbf{r}) $$

where *A*(**r**,**r** ^∗^) is the potential at **r** ^∗^ generated by an infinitesimal ’on’ source at **r**, and *H*(*x*) is the Heaviside step function (*H*(*x*) = 0for *x* < 0 and *H*(*x*) = 1 for *x* > 0). We computed *A*(**r**,**r** ^∗^) by using the BEM [22]. The strength of the ’on’ dipole layer follows from the observation that the activation wave front within the myocardium generates a potential jump of *σ*_*i*_/(*σ*_*i*_ + *σ*_*e*_)times the amplitude of the TMP [34].

The inverse problem in terms of the EDL source model for the depolarization phase entails finding the values of *τ*(**r**) at discretization nodes at the myocardial surface that reproduce the recorded ECGs at the electrode positions. The Nijmegen implementation of the EDL-based inverse method calculates the heart activation pattern in a two-step procedure. The non-linear nature of equation (2) necessitates finding an initial estimate for the activation times. In the second step, this estimate is fine-tuned in a non-linear optimization procedure.

The initial estimate of the activation times is provided by the fasted route algorithm (FRA) [7]. In this algorithm, each node on the heart surface is considered as an initial focus. For each node, the corresponding activation times on the heart surface are computed assuming different propagation velocites transmuraly and along the myocardial surface. The BSPMs of all the resulting activation patterns are computed and compared to the measured BSPM. The top 2% of activation patterns with the best correlation values are selected. The activation pattern within that 2% that produces the smallest relative difference (RD) is selected as the initial estimation. In case of sinus rhythm, the activation sequence is assumed to result from an ensemble of multiple ectopic foci with activation times following the principle “first come, first served.” For further details on the corresponding scheme, the reader is referred to [6].

The second step in the inverse procedure is a non-linear Marquardt-Levenberg optimization [27]. As the EDL-based inverse problem is ill-posed, measurement and modeling noise will cause substantial deviations from the true solution. To prevent unrealistic activation patterns, second-order Tikhonov regularization is applied. In our implementation, we used the Laplacian of the activation times to drive the final solution toward a physiologically realistic smoothess [7].

Regarding computational costs of the proposed methodology, calculation of initial estimate took less than 3 min. The second step, the optimization, took another minute (the inverse experiments were run on a Mac Pro, 2.66 GhZ Quad-Core Intel Xeon with 16 GB memory).

### Qualitative measures

The AT distributions calculated with the EDL-based inverse procedure were compared to the simulated AT distributions used for the bidomain forward calculations. To assess the performance of the inverse procedure for different anisotropy ratios, the following measures were used: root mean square (RMS), correlation (COR) and RD value between simulated and reconstructed activation times. Also the RD and COR values between the simulated (FEM) and estimated (BEM) BSPMs were determined. The relative difference RD was defined as follows: the Frobenius norm of difference between the simulated and reconstructed signals relative to those of the simulated data. For the ectopic beats, the distances between the simulated and inversely recovered foci were calculated. For sinus rhythm, the number and locations of early excitation sites were assessed.

## Results

### Sinus rhythm

**Fig. 2:**
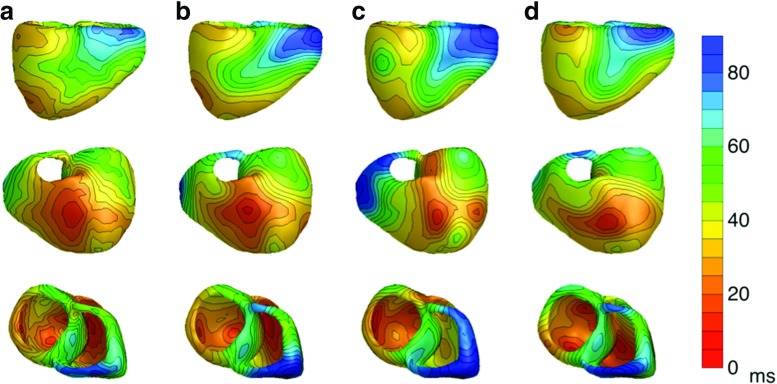
*Activation times in sinus rhythm:* The activation times in milliseconds on the ventricular heart surface. (Column **A**) The input activation times used in the bidomain model to construct the three different BSPM’s. This is the gold standard. (Column **B**) The activation times reconstructed with the EDL for the isotropic case. (Column **C**) The activation times for the anisotropic case with equal ratios for the intracellular and extracellular conductivities. (Column **D**) The activation times for the anisotropic case with unequal ratios for the intracellular and extracellular conductivities

For the BEM heart model, the activation pattern that was used as an input for the bidomain forward model shows four distinct sites of early activation (Fig. 2a). These are located on both sides of the septum near the apex, high on the left ventricular endocardial wall, and slightly later as an anterior epicardial breakthrough opposite to the septum. The input and inversely computed AT distributions for all conductivity setups are visualized in Fig. 2.

The activation times of the sinus rhythm are estimated reasonably well with the inverse procedure for all three types of FEM-simulated myocardial conductivities (isotropic, equal anisotropy and unequal anisotropy ratios). As expected, the isotropic case resulted in the best estimation and all four locations of early activation are found (Fig. 2b). This case has also the best RMS, COR, and RD values and shows the most similar activation pattern across the whole ventricular surface. The RMS, COR, and RD values for all three conductivity setups can be found in Table 2.

**Table 2:** Objective comparison measures of inverse calculations

Activation	Type of Myocardial	*λ*	RMS	COR	RD	COR	RD	DIST	DIFF 1st
Pattern	Conductivity		[ms]	ACT	ACT	BSPM	BSPM	[mm]	ACT [ms]
	Isotropic	4 ⋅ 10^− 6^	7.9	0.90	0.19	0.99	0.06	–	–
Sinus rhythm (1)	Anisotropic equal	5 ⋅ 10^− 6^	15.8	0.73	0.38	0.98	0.18	–	–
	Anisotropic unequal	9 ⋅ 10^− 6^	10.7	0.79	0.25	0.99	0.09	–	–
	Isotropic	4 ⋅ 10^− 6^	10.1	0.96	0.12	0.99	0.13	7.4	0
Left side of septum (2)	Anisotropic equal	5 ⋅ 10^− 6^	11.3	0.95	0.13	0.99	0.13	7.4	0
	Anisotropic unequal	4 ⋅ 10^− 5^	21.3	0.79	0.24	0.94	0.35	15.4	9
	Isotropic	3 ⋅ 10^− 6^	8.6	0.98	0.10	0.99	0.12	0	− 1
Right side of septum (3)	Anisotropic equal	5 ⋅ 10^− 6^	10.0	0.95	0.11	0.99	0.11	0	0
	Anisotropic unequal	5 ⋅ 10^− 5^	22.6	0.75	0.25	0.94	0.36	26.1	4
	Isotropic	5 ⋅ 10^− 6^	16.8	0.90	0.17	0.99	0.13	19.1	3
Base LV near septum (4)	Anisotropic equal	6 ⋅ 10^− 6^	15.3	0.90	0.16	0.99	0.11	19.1	2
	Anisotropic unequal	5 ⋅ 10^− 5^	18.0	0.88	0.18	0.96	0.28	19.1	8
	Isotropic	5 ⋅ 10^− 6^	19.9	0.84	0.20	0.99	0.14	23.1	14
Base RV near septum (5)	Anisotropic equal	7 ⋅ 10^− 6^	12.3	0.93	0.13	0.99	0.10	14.0	15
	Anisotropic unequal	5 ⋅ 10^− 5^	16.5	0.88	0.17	0.98	0.21	18.5	16
	Isotropic	4 ⋅ 10^− 6^	11.9	0.98	0.10	0.99	0.09	8.5	3
LV epicardial free wall (6)	Anisotropic equal	8 ⋅ 10^− 6^	16.1	0.94	0.14	0.99	0.09	11.1	5
	Anisotropic unequal	3 ⋅ 10^− 5^	17.1	0.94	0.15	0.97	0.22	24.9	14
	Isotropic	3 ⋅ 10^− 6^	11.8	0.97	0.10	0.99	0.09	7.7	− 1
LV endocardial free wall (7)	Anisotropic equal	8 ⋅ 10^− 6^	15.4	0.94	0.14	0.99	0.09	8.2	1
	Anisotropic unequal	3 ⋅ 10^− 5^	17.5	0.93	0.15	0.98	0.21	8.6	7
	Isotropic	3 ⋅ 10^− 6^	6.4	0.99	0.06	0.99	0.09	5.8	− 1
RV endocardial free wall (8)	Anisotropic equal	9 ⋅ 10^− 6^	11.4	0.97	0.10	0.99	0.27	5.8	− 1
	Anisotropic unequal	8 ⋅ 10^− 5^	46.2	0.31	0.42	0.94	0.37	34.0	28
	Isotropic	3 ⋅ 10^− 6^	6.4	0.99	0.06	0.99	0.09	0	− 4
RV epicardial free wall (9)	Anisotropic equal	2 ⋅ 10^− 5^	45.1	0.34	0.41	0.96	0.27	53.1	27
	Anisotropic unequal	7 ⋅ 10^− 5^	46.9	0.33	0.43	0.93	0.37	16.5	20.0

The RMS, COR and RD values are calculated between activation times on the heart surface, and the COR and RD values between the BSPM calculated with the EDL and calculated with the bidomain model. The activation patterns two to nine represented an ectopic focus starting in the indicated cardiac part. The abbreviations LV and RV are used to denote left and right ventricles, respectively

For the anisotropic cases, the results are expectedly worse, but the earliest sites of activation could still be determined (Fig. 2c, d ). For the case of equal anisotropy ratio, the right ventricle activates generally somewhat later. For the anisotropic case with unequal ratios, the early site of activation high on the left endocardial wall is not found.

### Ectopic foci

**Fig. 3:**
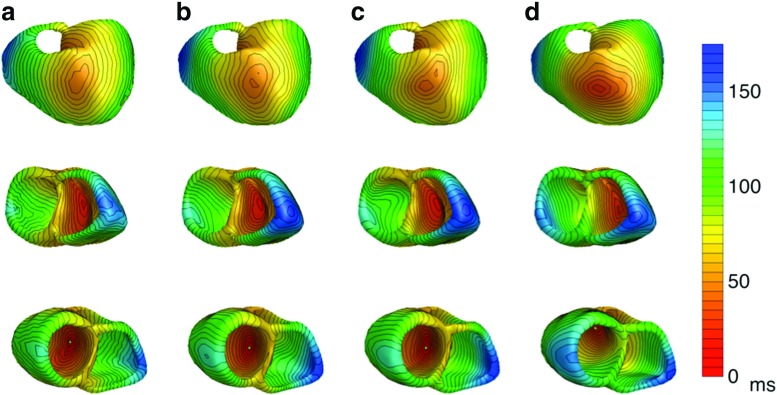
*Activation times for an ectopic beat with focus on the left ventricular septum:* The activation times in milliseconds on the ventricular heart surface. (Column **a**) The monodomain-simulated activation times used in the bidomain model to construct the three different BSPM’s. This is the gold standard. (Column **b**) The activation times reconstructed with the EDL for the isotropic case. (Column **c**) The activation times for the anisotropic case with equal ratios for the intracellular and extracellular conductivities. (Column **d**) The activation times for the anisotropic case with unequal ratios for the intracellular and extracellular conductivities. The earliest point of activation is indicated with a green dot

**Fig. 4:**
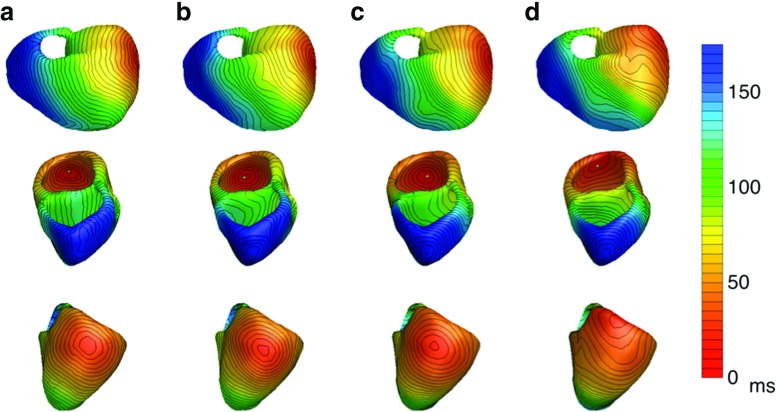
*Activation times for an ectopic beat with focus on endocardial side of left ventricular free wall:* The activation times in milliseconds on the ventricular heart surface. (Column **a**) The monodomain-simulated activation times used in the bidomain model to construct the three different BSPM’s. This is the gold standard. (Column **b**) The activation times reconstructed with the EDL for the isotropic case. (Column **c**) The activation times for the anisotropic case with equal ratios for the intracellular and extracellular conductivities. (Column **d**) The activation times for the anisotropic case with unequal ratios for the intracellular and extracellular conductivities. The earliest point of activation is indicated with a green dot

In total, eight ectopic beats were subject for the inverse reconstructions. The simulated and inversely estimated activation patterns for the septal focus in the left ventricle and for the endocardial focus on the left ventricular free wall are shown in Figs. 3 and 4, respectively. The activation patterns for the other ectopic beats can be found in the Supplementary material 1 (Fig. S1 till S6).

For all BSPMs simulated with an isotropic myocardium, the ectopic focus was found within a maximum distance of 23.1 mm. In six out of eight cases even within 10 mm, and in two cases the perfect ectopic focus location was found (Table 2). The inverse method was able to distinguish between endocardial and epicardial foci in all cases (panel A in Figs. 3 and 4, S1–S6). Moreover, the EDL-based procedure could differentiate between left and right septal foci and between left and right ventricular foci in the base area, which are known to be difficult for the ECG imaging [18].

For the anisotropic cases with equal anisotropy, seven out of eight ectopic foci were found within a distance of 20 mm. In four out of eight cases (activation patterns 2, 3, 7 & 8 in Table 2) even within 10 mm, in which for one case the perfect ectopic focus location was found. For the seven located foci, the reconstructed activation patterns were similar to the activation patterns from the isotropic case (Figs. 3b, 4b, 3c and 4c). Only for the ectopic sequence due to an epicardial focus on the right ventricular free wall (activation patterns 9 in Table 2), the inverse procedure resulted in 53.1 mm localization error. This is also represented in a low correlation value, high RD value and high RMS value (Table 2). Even in this situation, the focus was still correctly identified as epicardial.

Six out of eight reconstructions for the unequal anisotropy ratios case delivered a reasonable result: the focus was found within a distance of 26.1 mm. Only one focus (activation patterns 7 in Table 2) was found within 10 mm and none were precisely located. The ectopic beat due to an endocardial focus on the right ventricular free wall was incorrectly located on the epicardial side at a distance of 34.0 mm. (figure S5D) and the activation pattern produced with the inverse calculations had a high RMS value (Table 2). The activation pattern of the ectopic beat produced by a focus on the epicardial right ventricular free wall also had a high RMS value (Table 2), but the focus was correctly identified as epicardial and located within a distance of 16.5 mm (Fig. S6D).

### Ectopic foci right ventricular free wall

**Fig. 5:**
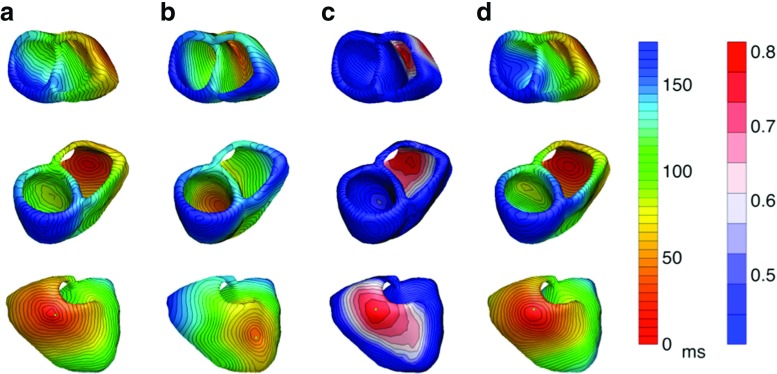
*Activation times ectopic beat with focus epicardial right ventricle:* (Column **a**) The activation times in milliseconds on the ventricular heart surface for The monodomain-simulated activation times used in the bidomain model to construct the three different BSPM’s. This is the gold standard. (Column **b**) The activation times reconstructed with the EDL for the anisotropic case with equal ratios for the intracellular and extracellular conductivities, and the initial solution with the best correlation and RD value. (Column **c**) The correlation values of the initial estimation for each node in the ventricular mesh computed with the FRA. The second best location can be seen in the bottom on the epicardial side of the right ventricular wall. (Column **d**) The activation times for the anisotropic case with equal ratios for the intracellular and extracellular conductivities, and the initial solution with the second best correlation and RD value. In columns **a**, **b**, and **d**, the earliest point of activation is indicated with a green dot

In three cases where the focus had epicardial breakthrough on the right ventricular wall (two instances for unequal anisotropy and one for equal anisotropy, Table 2), the overall activation pattern was not found (RMS error higher than 40 ms). In two out of these three cases, the focus was reconstructed at the correct side of the correct wall and in one case at the wrong side of the correct wall (see supplementary material: figures S6C and D, S5D). Further inspection showed that in these cases the initial estimate (see Section 2, inverse activation estimation) had identified two distinct patterns that explained the BSPMs almost equally well, one with the focus close to the correct location, and the other one with the focus far away. In these cases, the correlation between the ”measured” and model BSPM was slightly smaller for the pattern with the correct focus (for example, in one case: COR = 0.77 and RD = 0.70 vs. COR = 0.75 and RD 0.70). In Fig. 5c, the correlation values for the initial estimation are shown for the ectopic sequence due to an epicardial focus on the right ventricular free wall with equal anisotropy. Performing the inverse procedure using the second best initial estimate resulted in a good fit with the actual activation pattern in all these three cases (Table 3). The quantitative measures for the inverse reconstructions with the second best initial focus for all other activation patterns can be found in Supplementary material 2.

**Table 3:** Objective comparison measures of inverse calculations

Activation	Type of Myocardial	*λ*	RMS	COR	RD	COR	RD	DIST	DIFF 1st
Pattern	Conductivity		[ms]	ACT	ACT	BSPM	BSPM	[mm]	ACT [ms]
RV endocardial free wall (8)	Anisotropic equal	6 ⋅ 10^− 5^	14.6	0.96	0.13	0.87	0.50	6.3	4
RV epicardial free wall (9)	Anisotropic equal	1 ⋅ 10^− 5^	11.0	0.98	0.10	0.99	0.11	6.7	− 3
RV epicardial free wall (9)	Anisotropic unequal	6 ⋅ 10^− 5^	14.7	0.95	0.13	0.88	0.48	5.8	2

The RMS, COR, and RD values are calculated between activation times on the heart surface, and the COR and RD values between the BSPM calculated with the EDL and calculated with the bidomain model. The following activation patterns are presented with an index (8) endocardial free wall right ventricle, (9) epicardial free wall right ventricle

## Discussion

In the presented model-to-model study, we investigated the influence of myocardial anisotropy on the inverse solutions found with the EDL-based method, as developed in Nijmegen, The Netherlands [6, 22]. The cardiac anisotropy has been shown to have a great impact on formation of the excitation wave front and its inclusion is highly important in forward modeling [4, 5, 24, 26, 37, 45]. This study showed that the EDL-model used for the inverse calculations allows the reconstruction of the AT distributions and the distinction between endo- and epicardial sources, even though it ignores the unequal myocardial anisotropy ratio. The ectopic foci on the left and right side of the septum were also distinguished correctly. As the focus of present work was put on investigations of anisotropy role in the EDL-based inverse problem, the VCMs, electrode positions and conductivity values were kept the same for both forward and inverse computations, inevitably leading to certain model-to-model bias. However, the discretization of the used models and the numerical methods of electromagnetic fields calculation were distinct. Furthermore, the inverse modeling was accomplished without any knowledge of the underlying forward model parameters, which solidifies the obtained results. While clinical utility of the EDL-based approach was demonstrated in the valdiation study performed by Oosterhoff et al. [33], we believe that despite absence of outflow tracts in the considered heart model, the observations and conclusions about anisotropy role in the inverse computations would remain valid in other ventricular models as well. The EDL-base inverse can be applied clinically in a wide range of arrhythmias, such as WPW syndrome [16, 29] , Brugada syndrome [6] , and, as demonstrated in this study, focal VT. However, in its current implementation, it requires an electrically silent period in order to define the baseline. Consequently, application to arrhythmias like atrial fibrillation and reentrant VT will require further study.

Evaluation of the results for the isotropic case helps to distinguish the errors caused by the intrinsic ill-posedness of the inverse problem from those resulting from ignoring cardiac anisotropy. Expectedly, the isotropic case showed the best values of the considered quality measures for all activation sequences. Furthermore, as expected from the work of Geselowitz [14], the AT patterns reconstructed for the equal anisotropy ratios case were similar to those obtained from the isotropic simulations. In the isotropic case and equal anisotropy ratios case, all points of early activation were correctly reconstructed for sinus rhythm; for the ectopic activation sequences, all foci were correctly identified as either epicardial, endocardial or septal. Only one epicardial ectopic focus on the right ventricular free wall was reconstructed at 53.1 mm from the true location for the equal anisotropy ratios case. This was, however, due to an incorrect initial estimation (see Section 4, Initial estimation). For the basal part of the ventricles, the localization errors were somewhat higher than in other cases, although even for these foci a distinction was made between a right and left ventricular focus.

**Fig. 6:**
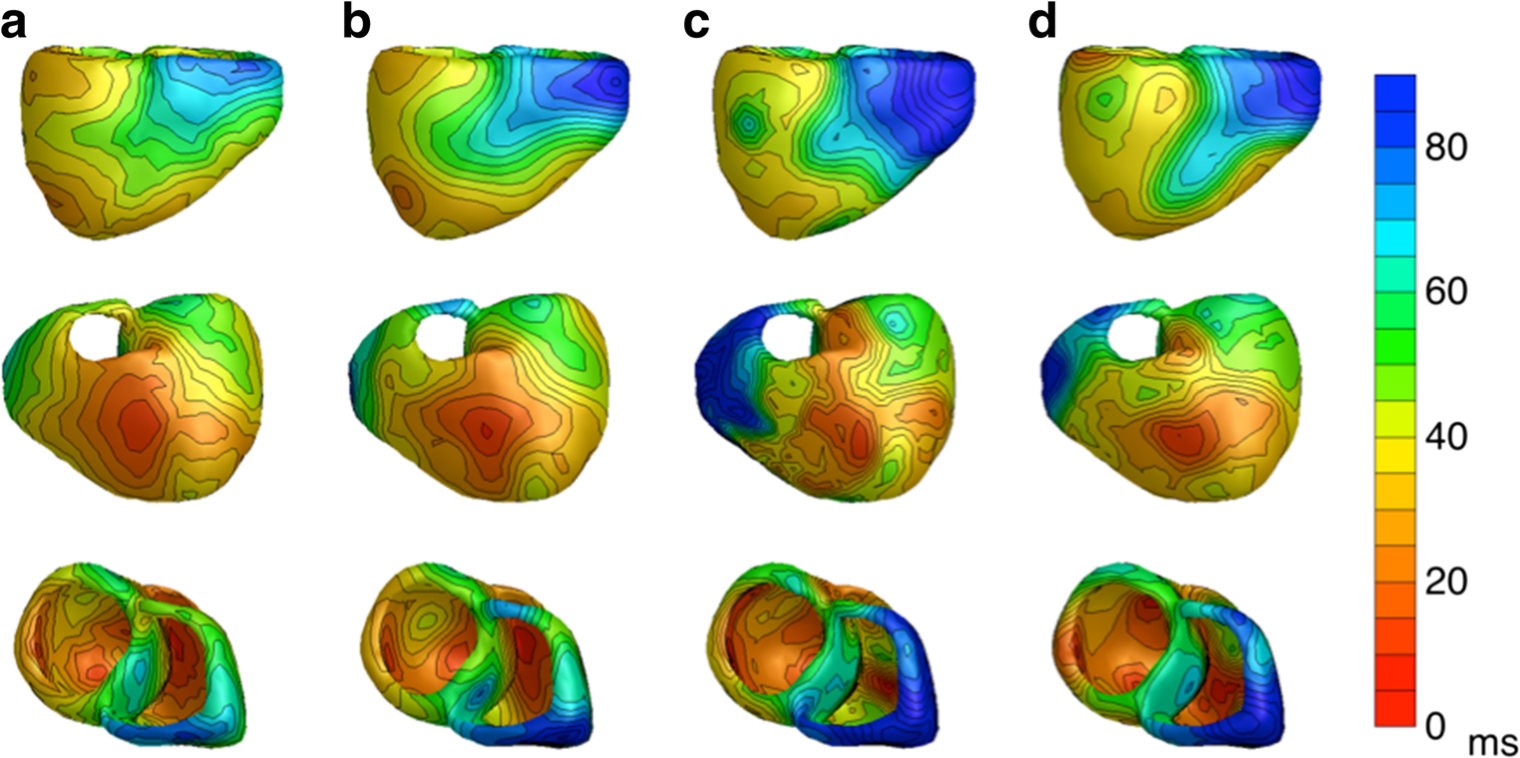
*Activation times sinus rhythm with less regularization:* The activation times in milliseconds on the ventricular heart surface. (Column **a**) The monodomain-simulated activation times used in the bidomain model to construct the three different BSPM’s. This is the gold standard. (Column **b**) The activation times reconstructed with the EDL for the isotropic case. (Column **c**) The activation times for the anisotropic case with equal ratios for the intracellular and extracellular conductivities. (Column **d**) The activation times for the anisotropic case with unequal ratios for the intracellular and extracellular conductivities

In the case of unequal anisotropy ratios, the inverse modeling errors were higher. For the simulated sinus rhythm, one point of early activation, situated endocardially on the left ventricular wall, was missed by the inverse procedure. However, this was obviously due to the chosen regularization (see Section 2, Inverse activation estimation), which may have been too strict. A less strict regularization that was applied after the real AT distributions were disclosed resulted in a reconstructed pattern that included all points of early activation (Fig. 6). Laplacian, as the regularization function, guards spatial smoothness of the solution and, thereby, controls its spatial variation. Due to this fact, applying a less strict regularization favored more spatial variation and enabled the reconstruction of the missed focal site that was otherwise suppressed by a too strict smoothness penalty. For the ectopic foci, the highest errors were observed for the two beats starting from the right ventricular free wall, similar to the epicardial focus of the equal anisotropy case. These incorrectly reconstructed activation patterns were mainly due to an incorrect initial estimation (see Section 4, Initial estimation).

### Previous work

Generally, the results from this study are in agreement with previous results for the inverse AT imaging method [28]. Previously, Modre et al. [28] have examined the influence of heart anisotropy on the reconstruction of AT for four different activation sequences (sinus rhythm, right-ventricular and septal pacing, sinus rhythm with pre-excitation). They showed that neglecting the heart anisotropy had a rather minor impact on the reconstruction quality of the AT distributions. With this regard, there are several differences between Modre et al. [28] and our study that are worth to be mentioned.

First of all, the initial AT distribution in the study conducted by Modre et al. [28] were simulated with a three-state cellular automaton [25] that does not include ion channel kinetics. In our work, we used a realistic state-of-the-art monodomain model for the forward calculations. Second, the previous work examined localization of a septal focus and an endocardial focus on the right ventricular free wall. Here, we extended the analysis by considering several ectopic foci on opposite sides of a myocardial wall. The results showed that the EDL-method was able to distinguish between endo- and epicardial sources, and left and right septal foci. Even for the unequal anisotropy case, the focus was found at the correct side of the septum. Third, an initial guess in Modre et al. [28] was provided by a critical point theorem [20], while in this study the FRA was used. Overall, the FRA has been proved to produce better results than the critical point theorem [32]. Finally, the forward and inverse calculations in Modre et al. [28] were performed on meshes with a spatial resolution much lower than those used in the present study, which might also be a reason for the increased localization errors in our work. In Wang et al. [47], some resolution guidelines were given for efficient solving of forward and inverse problems of ECG, and it was shown that an increasing spatial resolution on the heart can worsen the properties of the inverse problem approximation. Some more direct suggestions concerning the effect of mesh resolution on the ill-posedness of the ECG inverse problem were made by Hintermüller and colleagues [17].

### Initial estimation

As reported, there were three cases (one with equal anisotropy ratios and two with unequal anisotropy ratios) where the inverse procedure reconstructions resulted in a suboptimal AT distribution. Further analyses showed that the fastest route algorithm (FRA) found two initial estimates at distinct locations on the heart surface that performed virtually equally well. Using the second best initial estimate resulted in the correct localization. For the three activation sequences that were incorrectly reconstructed, the best initial focus location was situated low on the septum in the right ventricle. In Fig. 5b, a remnant of this initial estimation is still visible in the final solution for the equal anisotropy case. Selection of the second best initial estimate (Fig. 5c), at least more than 30 mm away from the best one, improved the final solutions drastically. All three activation patterns were reasonably reconstructed (Table 3 and Fig. 5d), although this was not always reflected in an improved correlation and RD value for the BSPM’s (Table 3). This analysis revealed that the considered inverse procedure heavily relies on the initial estimate and that the subsequent nonlinear routine minimizes the misfit between measured and simulated BSPMs for the local minimum delivered by the FRA. This was also confirmed by the observation that for the activation pattern with a focus at the right ventricular base near the septum (ectopic beat number 5), the distance error in equal anisotropy ratios case is lower than for the isotropic simulation. In presence of multiple initial estimates of equal quality, the proposed methodology would not provide a unique ablation target. From the locations that are equally likely according to the inverse procedure the most plausible one may be selected on anatomical and/or physiological knowledge, or during the ablation procedure several probable locations may be examined.

### Inclusion of anisotropy

Although the overall solution quality in the reconstruction of the AT distribution was high, improvements in the ability of inverse EDL-method to take cardiac anisotropy into account are desirable. One possibility is to use a more realistic distribution of conduction velocities in order to improve the initial estimate. Though in the clinical environment this information can only be measured indirectly, multiple simulations with modified parameters influencing the excitation propagation can provide some reliable estimates of conduction velocities for different heart regions. Furthermore, having in mind the need of keeping the inverse procedure as fast and as simple as possible, one can couple the BEM method for calculating the potentials arising in a volume conductor with a three-dimensional heart model including a rule-based fiber orientation [10] and a cellular automaton for simulating the candidate TMP distributions [16]. In [37], Potse et al. proposed to use the same, “compound,” anisotropy ratio for both intra- and extracellular spaces in combination with a dipole-source model. However, it remains unclear whether a rule-based introduced fiber orientation would have a considerable performance advantage over an isotropic model.

## Conclusions

We performed a realistic model-to-model study to examine the effects of neglecting heart anisotropy on AT imaging with the EDL-based non-linear inverse procedure and FRA for calculating the initial estimate. We forward calculated the BSPMs for a range of anisotropy ratios in the heart and compared the respective inverse reconstructions. Expectedly, isotropic and equal anisotropy ratio cases resulted in a better match between the simulated and recovered activation sequences. Based on the reconstructions for the sinus rhythm, we demonstrated that the choice of an optimal regularization parameter might be crucial for identifying all early activation sites for a multi-focal event. Furthermore, we showed that the cardiac anisotropy has a bigger effect on the reconstruction of some focal sources compared to others. However, despite higher distance errors for the unequal anisotropy ratios case, all but one considered foci were classified correctly based on criterion whether they originated from endo- or epicardium. We also showed that inclusion of a second best solution estimate as computed by FRA can substantially improve the localization of ectopic beats. The overall solution quality with the demonstrated ability of distinguishing between septal, endo- and epicardial sources revealed that the heart anisotropy might be neglected in the clinical applications of the considered inverse algorithm for noninvasive imaging of ventricular focal sources. Furthermore, the employed full-search was shown to be an effective tool for identification of possible solution uncertainties.

## Electronic supplementary material

Below is the link to the electronic supplementary material.

(DOC 3.64 MB)
