# Supplementary material for: Assessment of the equivalent dipole layer source model in the reconstruction of cardiac activation times on the basis of BSPMs produced by an anisotropic model of the heart
Source: Med Biol Eng Comput. 2017 Nov 13;56(6):1013–25. doi: 10.1007/s11517-017-1715-x (PMC5978848; doi:10.1007/s11517-017-1715-x)
Supplement: Supplementary file 1 — (DOC 3.64 MB) [file 11517_2017_1715_MOESM1_ESM.docx]

**Supplementary material 1**

**
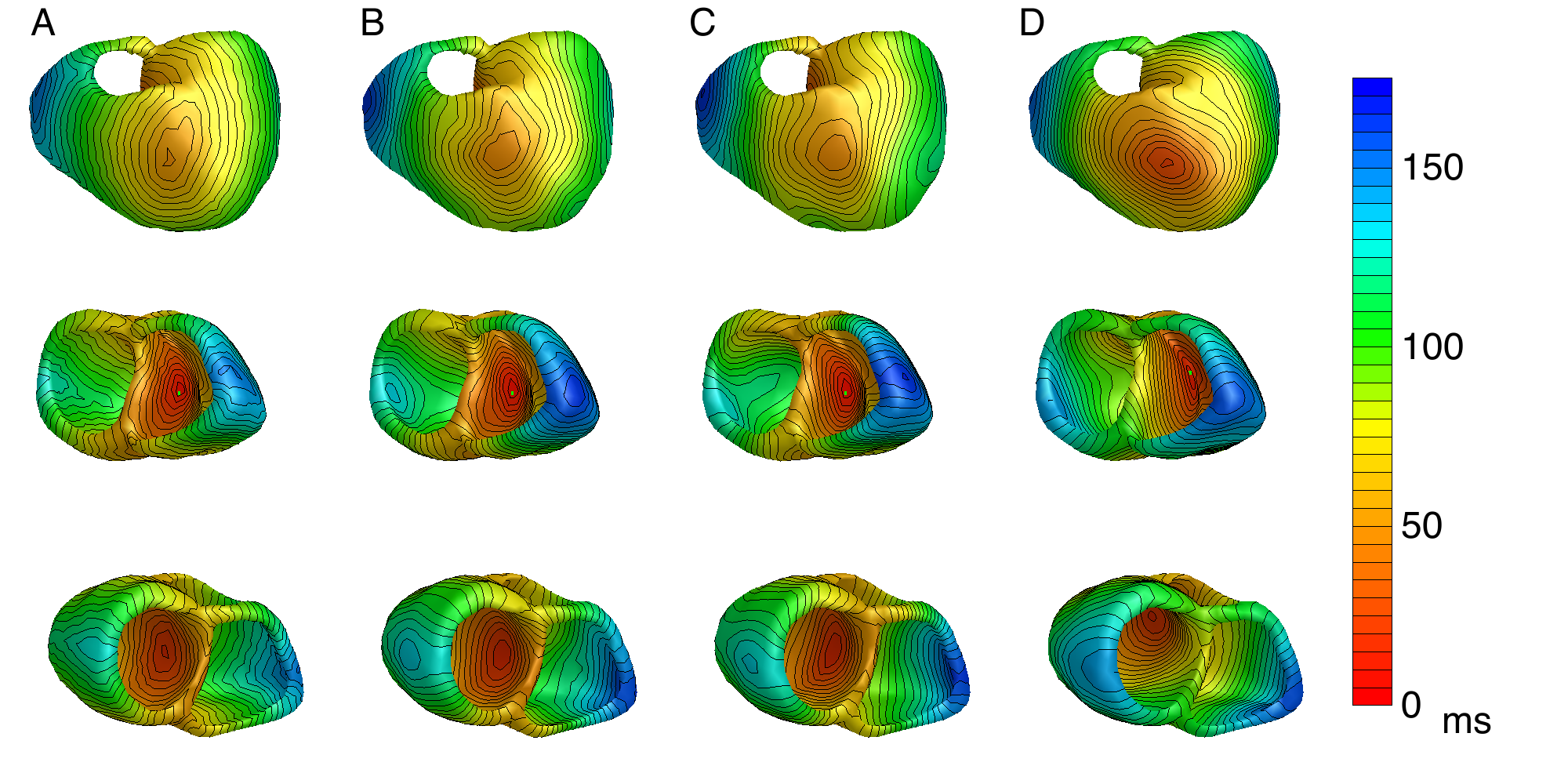
**

**Figure S1.** *Activation times for an ectopic beat with focus on the right ventricular septum.* The activation times in milliseconds on the ventricular heart surface. (Column A) The input activation times used in the bidomain model to construct the three different BSPM’s. This is the golden standard. (Column B) The activation times reconstructed with the EDL for the isotropic case. (Column C) The activation times for the anisotropic case with equal ratios for the intracellular and extracellular conductivities. (Column D) The activation times for the anisotropic case with unequal ratios for the intracellular and extracellular conductivities. The earliest point of activation is indicated with a green dot.


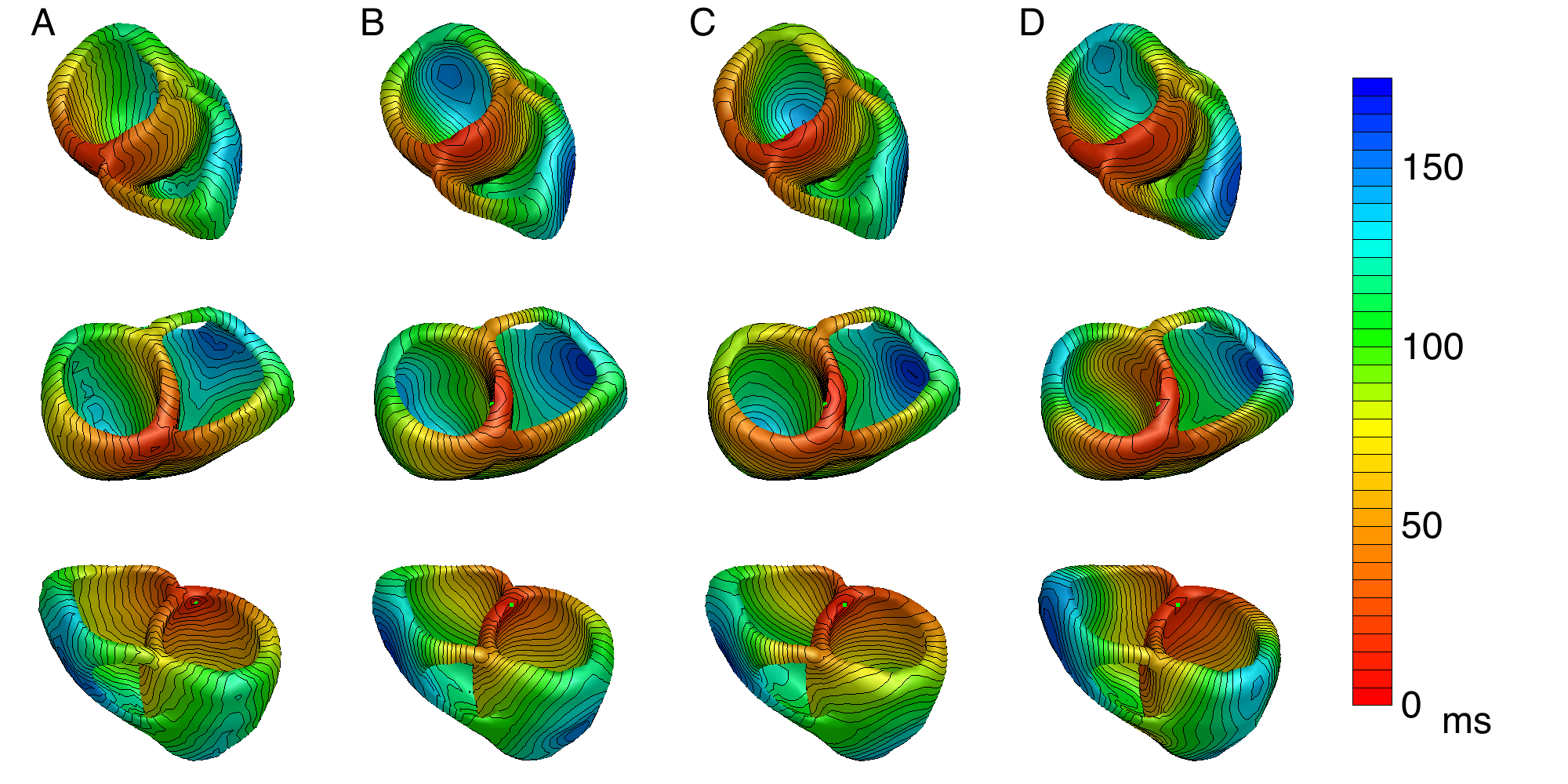


**Figure S2.** *Activation times for an ectopic beat with focus on base left ventricle, near septum.* The activation times in milliseconds on the ventricular heart surface. (Column A) The input activation times used in the bidomain model to construct the three different BSPM’s. This is the golden standard. (Column B) The activation times reconstructed with the EDL for the isotropic case. (Column C) The activation times for the anisotropic case with equal ratios for the intracellular and extracellular conductivities. (Column D) The activation times for the anisotropic case with unequal ratios for the intracellular and extracellular conductivities. The earliest point of activation is indicated with a green dot.


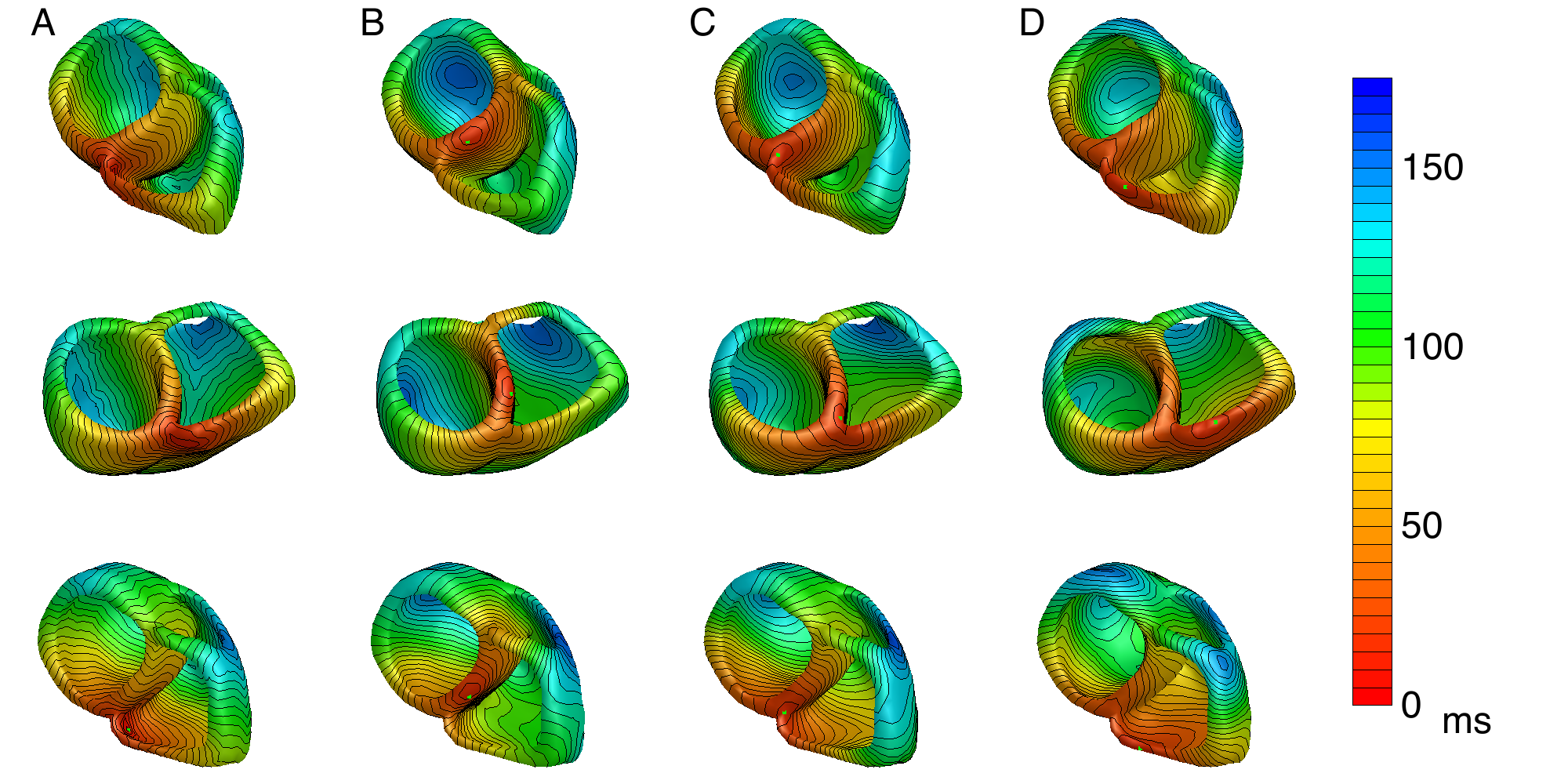


**Figure S3.** *Activation times for an ectopic beat with focus on base right ventricle, near septum.* The activation times in milliseconds on the ventricular heart surface. (Column A) The input activation times used in the bidomain model to construct the three different BSPM’s. This is the golden standard. (Column B) The activation times reconstructed with the EDL for the isotropic case. (Column C) The activation times for the anisotropic case with equal ratios for the intracellular and extracellular conductivities. (Column D) The activation times for the anisotropic case with unequal ratios for the intracellular and extracellular conductivities. The earliest point of activation is indicated with a green dot.


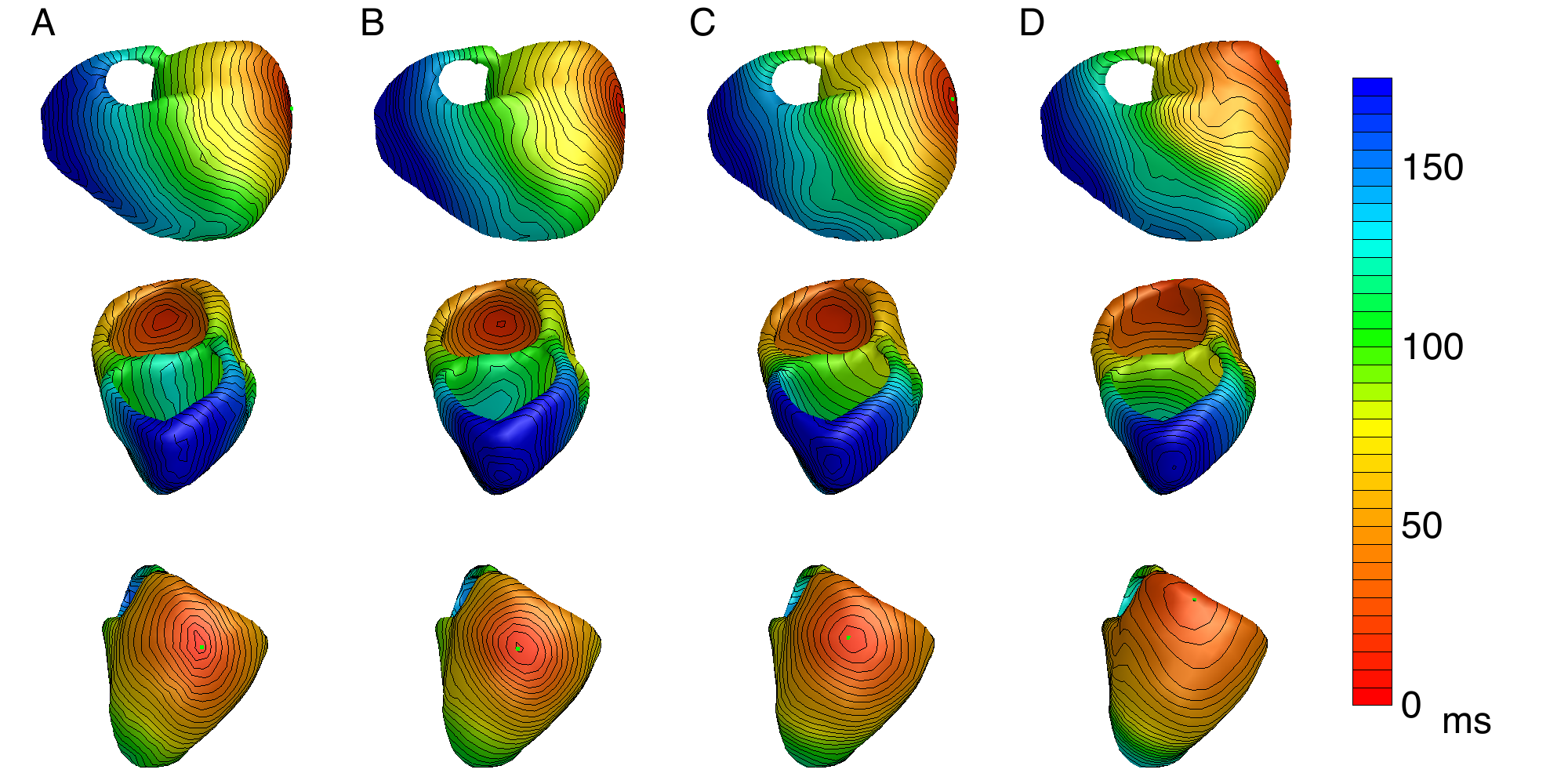


**Figure S4.** *Activation times for an ectopic beat with focus on epicardial side of left ventricular free wall.* The activation times in milliseconds on the ventricular heart surface. (Column A) The input activation times used in the bidomain model to construct the three different BSPM’s. This is the golden standard. (Column B) The activation times reconstructed with the EDL for the isotropic case. (Column C) The activation times for the anisotropic case with equal ratios for the intracellular and extracellular conductivities. (Column D) The activation times for the anisotropic case with unequal ratios for the intracellular and extracellular conductivities. The earliest point of activation is indicated with a green dot.


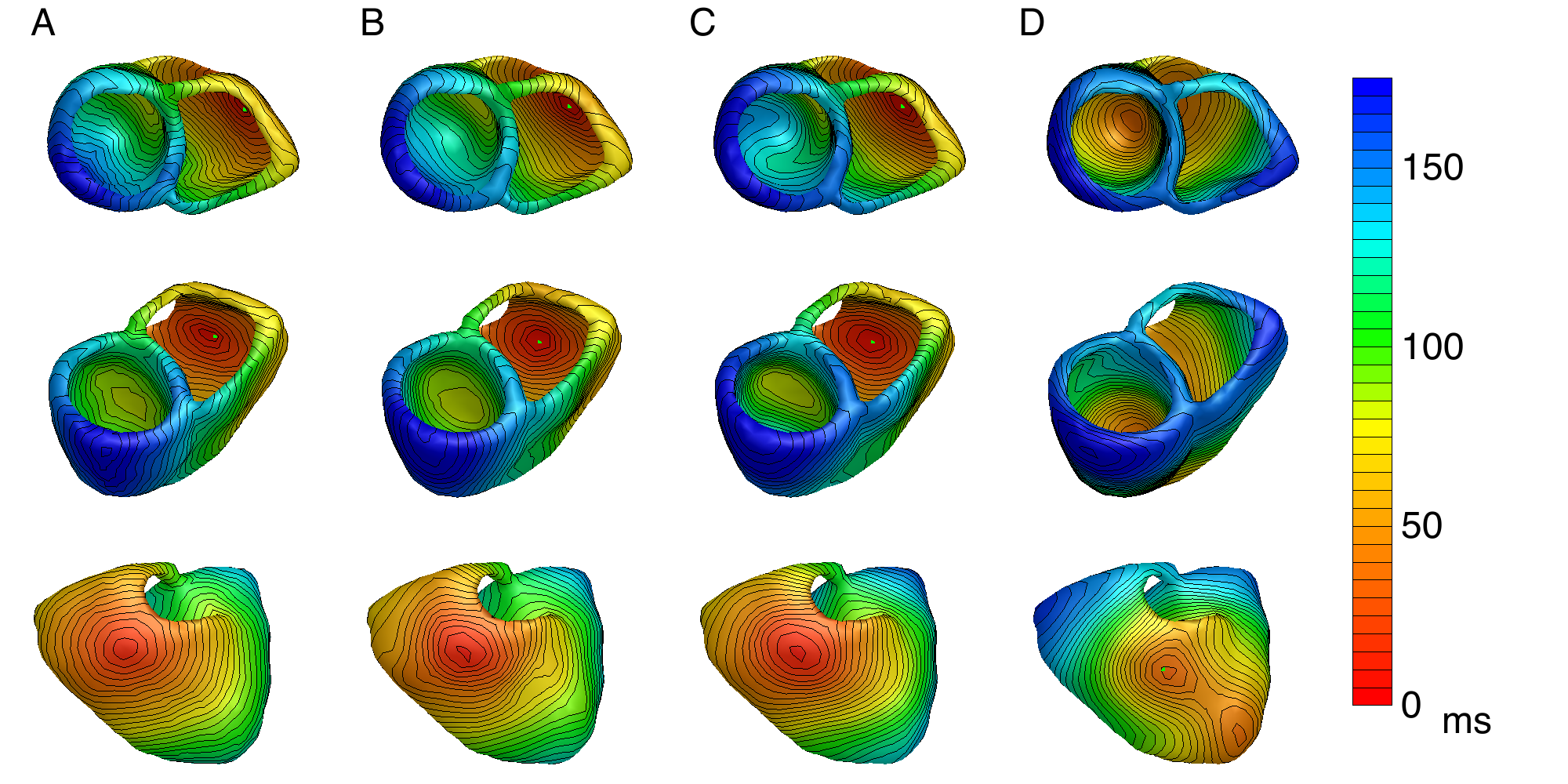


**Figure S5.** *Activation times for an ectopic beat with focus on endocardial side of right ventricular free wall.* The activation times in milliseconds on the ventricular heart surface. (Column A) The input activation times used in the bidomain model to construct the three different BSPM’s. This is the golden standard. (Column B) The activation times reconstructed with the EDL for the isotropic case. (Column C) The activation times for the anisotropic case with equal ratios for the intracellular and extracellular conductivities. (Column D) The activation times for the anisotropic case with unequal ratios for the intracellular and extracellular conductivities. The earliest point of activation is indicated with a green dot.


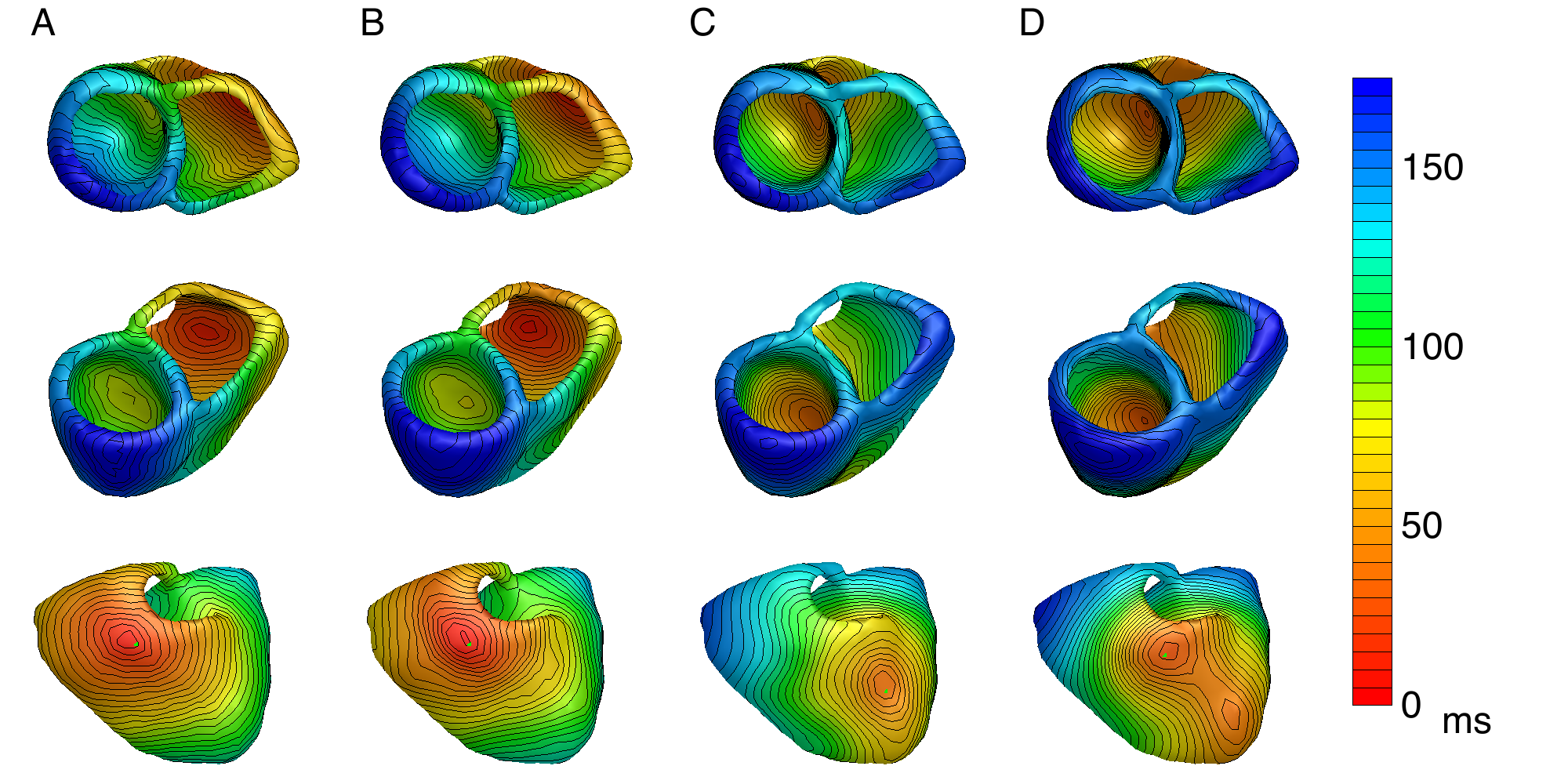


**Figure S6.** *Activation times for an ectopic beat with focus on epicardial side of right ventricular free wall.* The activation times in milliseconds on the ventricular heart surface. (Column A) The input activation times used in the bidomain model to construct the three different BSPM’s. This is the golden standard. (Column B) The activation times reconstructed with the EDL for the isotropic case. (Column C) The activation times for the anisotropic case with equal ratios for the intracellular and extracellular conductivities. (Column D) The activation times for the anisotropic case with unequal ratios for the intracellular and extracellular conductivities. The earliest point of activation is indicated with a green dot.

**Supplementary material 2**

**Table S1.** Objective comparison measures of inverse calculations. The RMS, COR and RD values are calculated between activation times on the heart surface, and the COR and RD values between the BSPM calculated with the EDL and calculated with the bidomain model. The following activation patterns are presented with an index: ectopic beats with focus on (2) left side of septum, (3) right side of septum, (4) base left ventricle near septum, (5) base right ventricle near septum, (6) epicardial free wall left ventricle, (7) endocardial free wall left ventricle, (8) endocardial free wall right ventricle, (9) epicardial free wall right ventricle.

| **Activation pattern** | **Type of myocardial conductivity** | $\lambda$ | **RMS [ms]** | **COR ACT** | **RD ACT** | **COR BSPM** | **RD BSPM** | **DIST [mm]** | **DIFF 1^st^ ACT [ms]** |
| --- | --- | --- | --- | --- | --- | --- | --- | --- | --- |
| 2 | Isotropic | 9•10^-6^ | 47.5 | -0.14 | 0.54 | 0.94 | 0.33 | 34.9 | 4 |
| 2 | Anisotropic equal | 1•10^-5^ | 45.5 | -0.11 | 0.52 | 0.95 | 0.32 | 36.6 | 4 |
| 2 | Anisotropic unequal | 3•10^-5^ | 47.1 | -0.13 | 0.54 | 0.94 | 0.35 | 33.3 | 7 |
| 3 | Isotropic | 1•10^-5^ | 54.5 | -0.30 | 0.61 | 0.95 | 0.33 | 34.6 | 4 |
| 3 | Anisotropic equal | 1•10^-5^ | 53.2 | -0.30 | 0.60 | 0.93 | 0.36 | 45.9 | 3 |
| 3 | Anisotropic unequal | 3•10^-5^ | 51.5 | -0.37 | 0.58 | 0.94 | 0.34 | 48.5 | 4 |
| 4 | Isotropic | 9•10^-6^ | 40.0 | 0.28 | 0.41 | 0.97 | 0.25 | 43.6 | 2 |
| 4 | Anisotropic equal | 9•10^-6^ | 13.0 | 0.92 | 0.13 | 0.99 | 0.13 | 5.6 | 3 |
| 4 | Anisotropic unequal | 6•10^-5^ | 22.0 | 0.83 | 0.23 | 0.90 | 0.44 | 20.2 | 7 |
| 5 | Isotropic | 9•10^-6^ | 31.0 | 0.54 | 0.32 | 0.98 | 0.22 | 53.6 | 18 |
| 5 | Anisotropic equal | 1•10^-5^ | 31.4 | 0.54 | 0.32 | 0.98 | 0.21 | 62.7 | 18 |
| 5 | Anisotropic unequal | 4•10^-5^ | 28.0 | 0.65 | 0.28 | 0.96 | 0.30 | 22.3 | 20 |
| 6 | Isotropic | 4•10^-6^ | 9.2 | 0.98 | 0.08 | 0.99 | 0.09 | 8.5 | 4 |
| 6 | Anisotropic equal | 9•10^-6^ | 12.4 | 0.96 | 0.11 | 0.99 | 0.09 | 11.1 | 8 |
| 6 | Anisotropic unequal | 9•10^-5^ | 31.2 | 0.75 | 0.27 | 0.90 | 0.45 | 24.8 | 11 |
| 7 | Isotropic | 4•10^-6^ | 9.0 | 0.98 | 0.08 | 0.99 | 0.09 | 7.7 | -1 |
| 7 | Anisotropic equal | 6•10^-6^ | 10.3 | 0.98 | 0.09 | 0.99 | 0.07 | 13.1 | 1 |
| 7 | Anisotropic unequal | 8•10^-5^ | 31.3 | 0.73 | 0.28 | 0.90 | 0.45 | 36.3 | 8 |
| 8 | Isotropic | 9•10^-6^ | 35.9 | 0.54 | 0.33 | 0.98 | 0.21 | 43.2 | 27 |
| 8 | Anisotropic equal | 2•10^-5^ | 42.1 | 0.37 | 0.39 | 0.97 | 0.24 | 37.7 | 29 |
| **8** | **Anisotropic unequal** | **6•10^-5^** | **14.6** | **0.96** | **0.13** | **0.87** | **0.50** | **6.3** | **4** |
| 9 | Isotropic | 1•10^-5^ | 37.6 | 0.50 | 0.34 | 0.98 | 0.22 | 46.0 | 21 |
| **9** | **Anisotropic equal** | **1•10^-5^** | **11.0** | **0.98** | **0.10** | **0.99** | **0.11** | **6.7** | **-3** |
| **9** | **Anisotropic unequal** | **6•10^-5^** | **14.7** | **0.95** | **0.13** | **0.88** | **0.48** | **5.8** | **2** |
